# *De Novo* Regulatory Motif Discovery Identifies Significant Motifs in Promoters of Five Classes of Plant Dehydrin Genes

**DOI:** 10.1371/journal.pone.0129016

**Published:** 2015-06-26

**Authors:** Yevgen Zolotarov, Martina Strömvik

**Affiliations:** Department of Plant Science, Macdonald Campus, McGill University, 21111 Lakeshore Road, Sainte-Anne-de-Bellevue, QC, H9X 3V9, Canada; Purdue University, UNITED STATES

## Abstract

Plants accumulate dehydrins in response to osmotic stresses. Dehydrins are divided into five different classes, which are thought to be regulated in different manners. To better understand differences in transcriptional regulation of the five dehydrin classes, *de novo* motif discovery was performed on 350 dehydrin promoter sequences from a total of 51 plant genomes. Overrepresented motifs were identified in the promoters of five dehydrin classes. The K_n_ dehydrin promoters contain motifs linked with meristem specific expression, as well as motifs linked with cold/dehydration and abscisic acid response. KS dehydrin promoters contain a motif with a GATA core. SK_n_ and Y_n_SK_n_ dehydrin promoters contain motifs that match elements connected with cold/dehydration, abscisic acid and light response. Y_n_K_n_ dehydrin promoters contain motifs that match abscisic acid and light response elements, but not cold/dehydration response elements. Conserved promoter motifs are present in the dehydrin classes and across different plant lineages, indicating that dehydrin gene regulation is likely also conserved.

## Introduction

Plants have developed specific mechanisms that allow them to prepare for and survive drastic changes in their environment. One of the better-studied mechanisms is cold acclimation, which allows plants to develop freezing tolerance [1,2]. During exposure to low non-freezing temperatures gene expression is modulated and numerous solutes, known as osmoprotectants, and protective proteins accumulate in plant tissues. Dehydrins or dehydration proteins, (DHN) belong to group II LEA (late embryogenesis abundant) proteins. They are often found among those protective proteins and they are ubiquitous in transcriptomes of plants under osmotic stress, such as cold, drought and high salinity [3–7]. All dehydrins contain a 15 amino acid K-segment, rich in lysine residues, represented by EKKGIMDKIKEKLPG conserved sequence [8]. The K-segment forms an amphipathic α-helix that allows dehydrins to stabilize plant membranes and proteins during dehydration stresses [9–12]. In addition to the K-segment, dehydrins can contain a Y-segment (T/VDEYGNP) and an S-segment (3+ serines) [13]. The S-segment is thought to be involved in ion binding and dehydrin phosphorylation, which induces a conformational change in dehydrins [14,15]. Currently, the function of the Y-segment is unknown. The dehydrins are categorized into 5 subclasses (K_n_, KS, SK_n_, Y_n_K_n_ and Y_n_SK_n_) based on the presence and location of the 3 conserved segments [13]. Members of each subclass are expressed in response to a different set of stimuli. However, there is no clear link between subclass types and expression triggers [16].

As defined by Close, 1997, dehydrins must contain a K-segment. According to that definition, dehydrins are only found in plants. There are other proteins described as dehydrins, for example from *Escherichia coli* (GenBank: AAB18249.1), a fungus *Pneumocystis carinii* (GenBank: CAC43457.1) [4] or from whitish truffle *Tuber borchii* (GenBank: ABC33908.1) [17]. However, these proteins do not contain the K-segment or any of the other conserved dehydrin segments and therefore should not be considered to be proper dehydrins.

Stress response in plants can be regulated in an abscisic acid (ABA) dependent and/or independent manner [18]. Multiple transcription factors, such as C-repeat binding factor/dehydration responsive element binding protein (CBF/DREB) and ABA response element binding protein (AREB), participate in water stress response, by binding to *cis*-regulatory elements in the promoters of their respective regulons. The CBF1-3 are transcription factors that participate in ABA independent cold and dehydration induced gene expression [19] and they bind a C-repeat (CRT) *cis*-regulatory element core (CCGAC), also known as dehydration response element (DRE). Members of the CBF regulon include well-studied *A*. *thaliana* genes, such as LTI78/COR78 [20], COR15A and COR47 (an SK_n_ dehydrin) [21]. However, not all members of the CBF regulon have the CRT *cis*-regulatory element in their promoters [22], hence there are yet undiscovered motifs that are involved in cold and drought response. Numerous transcription factors participate in the ABA dependent stress response and they bind several *cis*-regulatory elements with a TACGTG core [23]. Many members of the CBF regulon are also upregulated in response to drought and ABA exposure, demonstrating a cross-talk between stress-induced pathways [24]. For example, in barley (*Hordeum vulgare* L.), a K_n_ dehydrin is strongly upregulated in response to cold, dehydration and ABA, and its promoter contains CRT and abscisic acid response elements (ABREs) *cis*-regulatory elements, whereas a barley SK_n_ dehydrin, whose promoter contains multiple CRTs and no ABREs is only weakly upregulated in response to ABA, but shows a significant upregulation in response to cold [3]. The expression of CBFs, and, in turn, their regulons, is modulated by photoperiod through phytochrome B and phytochrome-interacting factors [25,26].

In this study, we tested whether the different classes of dehydrin genes house specific and conserved *cis*-regulatory elements in their promoters that could contribute to gene characterization. *De novo* motif discovery, a computational approach to identify statistically overrepresented sequence motifs within a promoter sequence, was used to analyze a total of 350 dehydrin promoters. For each of the five dehydrin classes, statistically significant motifs were identified, and matched to experimentally validated *cis*-regulatory elements known from literature. Motifs linked to ABA-dependent and ABA-independent stress response pathways were detected in the promoters of dehydrin genes from various, distant plant lineages, which indicates that the stress response pathways regulating dehydrin expression are conserved.

## Methods

### Plant genomes used in the computational analyses

Permission to use data from genomes that are not published was obtained from members of sequencing consortia, where stated. In other cases, published data was used.

The following genome sequences were obtained from Phytozome v10 (http://phytozome.jgi.doe.gov) [27] using BioMart [28]: *Amborella trichopoda* [29], *Aquilegia coerulea* (*Aquilegia coerulea* Genome Sequencing Project, http://www.phytozome.net/, permission obtained from Dr. Scott Hodges), *Arabidopsis halleri* (*Arabidopsis halleri* v1.1, DOE-JGI, http://www.phytozome.net/ahalleri), *Arabidopsis lyrata* [30], *Arabidopsis thaliana* [31], *Boechera stricta* (*Boechera stricta* v1.2, DOE-JGI, http://www.phytozome.net/bstricta), *Brachypodium distachyon* [32], turnip mustard (*Brassica rapa* L.*)* [33,34], papaya (*Carica papaya)* [35], *Capsella grandiflora* and *Capsella rubella* [36], clementine (*Citrus clementina)* and, sweet orange (*Citrus sinensis)* [37], cucumber (*Cucumis sativus)* (permission obtained from Dr. Yiqun Weng), *Eucalyptus grandis* [38], *Eutrema salsugineum* (formerly *Thellungiella halophila*) [39], strawberry (*Fragaria vesca)* [40], soybean (*Glycine max)* [41], cotton (*Gossypuim raimondii)* [42], flax (*Linum usitatissimum)* [43], apple (*Malus domestica)* [44], cassava (*Manihot esculenta)* [45], barrel medic (*Medicago truncatula*) [46], monkey flower (*Mimulus guttatus*) [47], rice (*Oryza sativa*) [48], swtichgrass (*Panicum virgatum v1*.*0*, *DOE-JGI*, *http://www.phytozome.net/pvirgatum*), common bean (*Phaseolus vulgaris* L.*)* [49], moss (*Physcomitrella patens)* [50], peach (*Prunus persica)* [51], poplar (*Populus trichocarpa)* [52], castor bean (*Ricinus communis)* [53], foxtail millet (*Setaria italica)* [54], Shrub willow (*Salix purpurea* v1.0, DOE-JGI, http://phytozome.jgi.doe.gov/pz/portal.html#!info?alias=Org_Spurpurea), tomato (*Solanum lycopersicum)* [55], potato (*Solanum tuberosum)* [56], greater duckweed (*Spirodela polyrhiza*) [57], cocoa (*Theobroma cacao*) [58], grape (*Vitis vinifera)* [59], maize (*Zea mays)* [60].

The following genomes were obtained from other sources: kiwifruit (*Actinidia chinensis*) [61], sugar beet (*Beta vulgaris*) [62]; pigeonpea (*Cajanus cajan*) [63] http://www.icrisat.org/gt-bt/iipg/genomedata.zip; pepper (*Capsicum annuum*) [64]; chickpea (*Cicer arietinum*) [65] http://www.icrisat.org/gt-bt/ICGGC/genomedata.zip; *Lotus japonicus* [66] ftp://ftp.kazusa.or.jp/pub/lotus/lotus_r2.5/; banana (*Musa acuminata*) [67]; *Oryza brachyantha* [68]; date palm (*Phoenix dactylifera*, Draft Sequence Version 3) [69] http://qatar-weill.cornell.edu/research/datepalmGenome/download.html; Norway spruce (*Picea abies*) [70]; loblolly pine (*Pinus taeda*) [71].

### Identification of dehydrin genes

A custom solution was used to identify all dehydrin genes found in the plant genomes described above. Amino acid sequences of several known dehydrins were obtained from NCBI GenBank [72] and sequences of their K-segment were used to populate a seed FASTA file. A Python script was written that used Biopython [73] Motif module to scan all amino acid sequence for proteins containing a sequence similar to the K-segment, based on its position frequency matrix (PFM). After each round of search new K-segment sequences were added to the original FASTA file. The Y-segment sequence file was constructed in a similar manner using identified dehydrin protein sequences. Identified dehydrins were categorized based on the occurrence of conserved segments using either their PFMs (K- and Y-segments) or a regular expression that described a simpler S-segment. All identified dehydrins were divided into five categories: K_n_, KS, SK_n_, Y_n_K_n_, Y_n_SK_n_ and 1000 bps upstream of the transcription start site (where data was available, otherwise upstream from the start site) were obtained from Phytozome BioMart or they were directly extracted from the genomes using custom scripts. *Oxytropis arctobia* and *Oxytropis splendens* KS dehydrin gene sequences were obtained from NCBI GenBank (accessions: AEV59613 and AEV59617, respectively [6]). 1000 bp of *O*. *arctobia* and *O*. *splendens* promoters were obtained by amplifying GenomeWalker libraries and sequencing PCR products (Zolotarov et. al., unpublished).

To validate that the identified genes can actually be considered dehydrins, phmmer, as implemented on the HMMER web server [74] was used to search sequences on UniProt Knowledgebase [75] that have a significant similarity to putative dehydrins discovered using our custom method. The top ten significant hits were taken for each putative dehydrin and their domain annotation was extracted. Additionally, Needleman-Wunsch [76] pairwise alignment was used to compare 15 putative KS dehydrin to a known *Arabidopsis* KS dehydrin (AT1G54410). The closest match to every putative dehydrin in the NCBI GenBank non-redundant database was searched for using BLAST [77].

All the scripts and sequence data used in this paper are available from https://github.com/zolotarov/dehydrin_promoters

### Intrinsic disorder and hydrophilicity analysis

The identified dehydrin sequences were compared for intrinsic disorder and hydrophilicity with random plant protein sequences to assess the classification as a dehydrin. To calculate the grand average of hydrophilicity, Biopython ProtParam module was used. To calculate disorder proportion, IUPred [78] scores were calculated for each amino acid. The proportion of amino acids with the score above 0.5 (indicating disorder) was calculated. Statistical comparison was performed using t-test implemented in the scipy library [79,80]. The same number of random protein sequences was obtained for each species as the number of dehydrins used in this study. The sequences were downloaded using NCBI Entrez Direct E-utilities [81].

### De novo motif discovery

Motifs were discovered using MEME v4.9.1 [82], Seeder v0.01 [83] and Weeder v1.4.2 [84], and using the five sets of sequences as separate input. Significant motifs were selected based on following parameters: E-value ≤ 0.05 for MEME, Q-value ≤ 0.01 for Seeder and the top 3 motifs recommended by Weeder adviser. All promoters that were available through Phytozome BioMart from all species included in the analyses, was used as a background set (a total of 1029220 promoters). A separate parser was written to extract significant PFMs from result files produced by each program. The PFMs produced for each dehydrin class were entered into the STAMP [85] website to group matrices by similarity and to identify significant (E-value ≤ 0.05) matches in PLACE [23]. A representative member from a tree node of matrices grouped by similarity was selected and its sequence logo was generated using WebLogo 3.3 [86].

## Results and Discussion

In order to further understand how different dehydrins are regulated in response to environmental stress, motifs corresponding to conserved *cis*-regulatory elements were detected in the upstream regions of dehydrin genes in all five subclasses. Dehydrin proteins are by nature unstructured, and a custom identification strategy was employed to retrieve as many dehydrin genes with up to 1000 bp upstream region as possible. In total, 340 dehydrin promoters of size 1000 bp and eight dehydrin promoters of shorter length were retrieved from 51 plant genome sequences. In addition, two promoters from dehydrin genes isolated from two *Oxytropis* species were also included (Table 1, S1 Table). Out of the queried genomes, 10 were from monocotyledonous plants, 37 from dicotyledonous plants, one from a basal angiosperm (*Amborella trichopoda*), two from gymnosperms (*Picea abies* and *Pinus taeda*) and one from moss (*Physcomitrella patens*). The 350 sequences identified were confirmed to also match annotated dehydrins. For 330 out of 350 sequences, at least half of the top ten significant hits had “Dehydrin” as domain annotation. For the remaining 20 putative dehydrins, less than half of the top ten significant hits carried that annotation. Out of those, three were annotated as either a dehydrin or similar to dehydrin on NCBI GenBank and two were annotated as having a dehydrin domain on UniProtKB. The rest of the sequences were all short putative KS dehydrins. In these cases, all significant phmmer hits were analyzed. From 14.2% to 30.1% of significant hits had “Dehydrin” domain architecture, for sequences with lowest and highest number of significant hits with “Dehydrin” annotation, respectively. The rest of significant hits had no architecture annotation. When Needleman-Wunsch pairwise alignment was used to compare 15 putative KS dehydrin to a known *Arabidopsis* KS dehydrin (AT1G54410), sequence similarities ranged from 59.0% to 98.0%. This evidence supports the notion that the sequences extracted for the analyses can be classified as dehydrins.

**Table 1:** Number of analyzed dehydrin promoters per species.

Species	K_n_	KS	SK_n_	Y_n_K_n_	Y_n_SK_n_	Total
*Actinidia chinensis*	2	1	4	0	2	9
*Amborella trichopoda*	0	1	2	0	0	3
*Aquilegia coerulea*	1	1	2	1	1	6
*Arabidopsis halleri*	0	0	3	1	2	6
*Arabidopsis lyrata*	1	1	5	1	2	10
*Arabidopsis thaliana*	1	1	4	1	3	10
*Beta vulgaris*	0	0	2	0	2	4
*Boechera stricta*	1	1	4	0	3	9
*Brachypodium distachyon*	0	1	4	0	5	10
*Brassica rapa*	0	1	6	0	4	11
*Cajanus cajan*	0	1	2	1	1	5
*Capsella grandiflora*	1	1	6	0	1	9
*Capsella rubella*	1	1	4	0	3	9
*Capsicum annuum*	0	2	2	0	5	9
*Carica papaya*	0	1	1	0	2	4
*Cicer arietinum*	0	2	1	1	1	5
*Citrus clementina*	1	0	1	0	2	4
*Citrus sinensis*	0	1	2	0	3	6
*Cucumis sativus*	0	1	1	0	2	4
*Eucalyptus grandis*	1	0	1	0	4	6
*Eutrema salsugineum*	0	1	4	1	3	9
*Fragaria vesca*	0	0	1	0	5	6
*Glycine max*	0	4	1	2	3	10
*Gossypium raimondii*	1	0	3	1	3	8
*Linum usitatissimum*	0	1	6	2	2	11
*Lotus japonicus*	0	0	2	1	1	4
*Malus domestica*	0	0	2	2	5	9
*Manihot esculenta*	0	2	1	0	2	5
*Medicago truncatula*	0	1	1	1	1	4
*Mimulus guttatus*	0	1	1	0	2	4
*Musa acuminata*	0	2	1	0	1	4
*Oryza brachyantha*	0	1	1	0	5	7
*Oryza sativa*	0	1	1	0	6	8
*Panicum virgatum*	0	3	2	0	5	10
*Phaseolus vulgaris*	0	1	1	1	1	4
*Phoenix dactylifera*	2	1	2	0	1	6
*Physcomitrella patens*	4	0	0	0	0	4
*Picea abies*	4	0	9	0	0	13
*Pinus taeda*	8	0	10	0	0	18
*Populus trichocarpa*	3	1	1	0	2	7
*Prunus persica*	0	0	2	2	2	6
*Ricinus communis*	0	1	1	0	3	5
*Salix purpurea*	5	1	2	2	0	10
*Setaria italica*	0	0	2	0	5	7
*Solanum lycopersicum*	0	0	1	0	4	5
*Solanum tuberosum*	0	1	1	0	3	5
*Sorghum bicolor*	0	1	1	0	3	5
*Spirodela polyrhiza*	1	0	0	0	0	1
*Theobroma cacao*	1	1	1	0	2	5
*Vitis vinifera*	0	0	0	0	2	2
*Zea mays*	0	2	2	0	3	7
*Oxytropis splendens* *	–	1	–	–	–	1
*Oxytropis arctobia* *	–	1	–	–	–	1
**Total**	39	47	120	21	123	350

* Only one promoter was obtained per species, using genome walking.

### Biochemical properties of dehydrins

Dehydrins are known to be intrinsically disordered and hydrophilic [87], making it difficult, if not impossible, to identify them by overall sequence homology. These properties are, however, important for their hypothesized function in protein stabilization through interaction with water molecules, as well as for their subcellular location in the cytosol and the nucleus and not within membranes [87,88]. To assess the identification of the dehydrins included in this study, the grand average of hydropathicity (GRAVY, [89]) and the proportions of amino acids in the disordered regions were compared between dehydrins and random plant proteins. It was found that the 350 dehydrin amino acid sequences analyzed, were significantly more hydrophilic than 350 random plant protein sequences (GRAVY -1.3470 for dehydrins, -0.2938 for random plant proteins, p-value < 0.001). The level of structural disorder indicated that in the dehydrins analyzed, the average proportion of amino acid sequences in the state of disorder was 99.32% compared to 15.95% in random plant proteins (p-value < 0.001).

### Promoters of KS dehydrins have one conserved GATA motif

In total, 47 KS dehydrin promoters were included in the *de novo* motif discovery (Table 1). Using the *de novo* motif discovery tool Seeder [83,90], one single putative conserved regulatory motif was discovered in all 47 promoter sequences (Motif 1, Table 2, S2 Table). A similar motif was also discovered with Weeder [84]. The KS dehydrins are known to be expressed in response to cold and dehydration, as well as being constitutively expressed [6,91–93]. Although the single identified overrepresented motif in KS dehydrin promoters does not directly match any typical cold or dehydration-related *cis*-regulatory elements in the PLACE database [23], it does match two motifs involved in light regulation and one involved in sugar regulation (Table 2): IBOXCORENT (I-box core) [94], REBETALGLHCB21 [95] and SREATMSD [96], respectively. These three experimentally validated motifs share four nucleotides (GATA).

**Table 2:** Selected *de novo* motifs found in KS dehydrin promoters and their putative function identified through PLACE database.

	Match in PLACE^5^			
*De novo* motif	Sequence	Name	E-value^6^	Function
1. Seeder^1^ AWTCGGATAA^**2**^ (47/47^**3**^, 8.8e-07^4^)	GATAAGR	IBOXCORENT	1.4e-08	Found in light-responsive conserved DNA modular arrays
	TTATCC	SREATMSD	1.2e-07	Sugar-repressive element (SRE) found in genes down-regulated after main stem decapitation
	CGGATA	REBETALGLHCB21	1.8e-07	Required for phytochrome regulation

^1^Number of the motif and the *de novo* discovery software that was used to locate that motif.

^2^Motif consensus sequence in IUPAC nucleotide code.

^3^Occurrence is the number of promoters containing a *de novo* motif out of the total number of promoters analyzed for a specific dehydrin class, presented in the parentheses.

^4^Siginificance of the motif, E-value calculated by MEME, Q-value calculated by Seeder, presented in the parentheses.

^5^PLACE matches were identified using STAMP, only significant matches with E-value < 0.05 are presented.

^6^E-value of the match with PLACE motif.

One of these motifs, the I-box (GATAAGR) can form a light-responsive conserved DNA modular array (CMA) together with a G-box (CACGTGGC) when located in close proximity to one another. In transgenic Arabidopsis and tobacco (*Nicotiana tabacum*) plants, the presence of this CMA in a promoter, drives GUS reporter gene expression when exposed to light. Interestingly, this expression seems to be mediated by phytochrome and cryptochrome photoreceptors [94].

Another of the motifs matching the motif discovered in the KS dehydrin promoters, the REBETALGLHCB21, also called REβ (CGGATA), was first identified in gibbous duckweed (*Lemna gibba*) [95]. It is involved in phytochrome-mediated repression of promoter activity in darkness, when located in close proximity with REα (AACCAA). Although REα was not identified as a significantly overrepresented motif, it is found in 26 out of the 47 KS dehydrin promoters analyzed. The GATA part of the REβ was shown to be absolutely necessary for darkness-induced repression [95]. Furthermore, in Arabidopsis, C-repeat (CCGAC, CRT)-linked cold and dehydration induced gene expression is mediated by phytochrome [25]. While CRT was not found to be significantly overrepresented within the set of KS dehydrin promoters, it is noteworthy that 27 out of the 47 KS dehydrin promoters contain one or more copies of CRT or its reverse complement. Sixteen of the promoters contain both REα and REβ.

The motif discovered in the KS dehydrin promoters also matched a sugar-repressive element, SREATMSD (TTATCC, SRE), shown to be involved in sugar mediated gene repression in Arabidopsis [96]. Sugars are known osmoprotectants that are produced by plants in response to cold [97]. One of the suggested roles of dehydrins is in the stabilization of protein conformation. Sugars, such as sucrose and trehalose, can replace water molecules on the surface of a protein and can thus conserve its conformation. This allows cells to restore their function after rehydration [98].

### Motifs discovered in promoters of K_n_ match abscisic acid and low temperature response elements

A total of 39 K_n_ dehydrin promoters were included in the *de novo* regulatory motif discovery analysis (Table 1). The K_n_ dehydrins are expressed in response to high salinity, abscisic acid (ABA), cold and dehydration [3,5,99,100]. A total of three putative regulatory motifs were identified in this set of promoters (Table 3, S2 Table)—two were discovered using MEME (Motif 2: GGCAGGAC/GTGGTGCC; and Motif 3: ATGTCGGC/GCCGACAT) and one using Seeder (Motif 4: TCGCCGACAT/ATGTCGGCGA). Motif 2 (GGCAGGAC) has a significant match to the SITEIIBOSPCNA (TGGTCCCAC) motif in the PLACE database. This motif is linked with meristematic tissue-specific gene expression in rice (*Oryza sativa*) [101] and it was found in 31 out of the 39 promoters. Motifs 3 and 4, found in all analyzed K_n_ dehydrin promoters, match DREDR1ATRD29AB motif (TACCGACAT) [102] and LTREATLTI78 (ACCGACA) [103], two low temperature response elements (LTREs) involved in cold response in *A*. *thaliana*. Additionally, Motif 3 matches an ABRE found in wheat and rice- ABREOSRAB21 (ACGTSSSC) [104]. The presence of both LTREs and an ABRE indicates that K_n_ dehydrins, similarly to SK_n_ and Y_n_SK_n_ dehydrins, could be expressed in ABA-dependent and independent manner in response to osmotic stresses.

**Table 3:** Selected *de novo* motifs found in K_n_ dehydrin promoters and their putative function identified through PLACE database.

	Match in PLACE^5^			
*De novo* motif	Sequence	Name	E-value^6^	Function
2. MEME^1^ GGCMCCAC^**2**^ (31/39^3^, 1.2e-06^4^)	TGGTCCCAC	SITEIIBOSPCNA	4.3e-07	Involved for meristematic tissue-specific expression in rice
3. MEME AYGTCGGY (39/39, 3.7e-05)	TACCGACAT	DREDR1ATRD29AB	6.4e-11	Response to drought, low temperature and high salinity. Bound by CBF1 in *Arabidopsis*
	ACCGACA	LTREATLTI78	5.5e-09	LTRE
	ACGTSSSC	ABREOSRAB21	3.3e-05	ABRE found in wheat and rice
4. Seeder WNRCCGACAT (39/39, 2.1e-05)	ACCGACA	LTREATLTI78	3.7e-07	LTRE
	TACCGACAT	DREDR1ATRD29AB	4.9e-08	Response to drought, low temperature and high salinity. Bound by CBF1 in *Arabidopsis*

^1^Number of the motif and the *de novo* discovery software that was used to locate that motif.

^2^Motif consensus sequence in IUPAC nucleotide code.

^3^Occurrence is the number of promoters containing a *de novo* motif out of the total number of promoters analyzed for a specific dehydrin class, presented in the parentheses.

^4^Siginificance of the motif, E-value calculated by MEME, Q-value calculated by Seeder, presented in the parentheses.

^5^PLACE matches were identified using STAMP, only significant matches with E-value < 0.05 are presented.

^6^E-value of the match with PLACE motif.

### SK_n_ dehydrins contain multiple cold/dehydration, abscisic acid and light regulated response elements

A total of 120 SK_n_ dehydrin promoters were analyzed (Table 1). Six *de novo* discovered putative regulatory motifs are presented in Table 4 and S2 Table. MEME and Seeder each discovered three motifs. The SK_n_ dehydrins are known to be expressed in response to cold, ABA, dehydration and salt [3,5,14,105]. Three out of six motifs (motifs 5–7) have matches in PLACE that are known ABREs. Motif 5 (CCACGTGTC/GACACGTGG) matches ABREs from wheat (*Triticum aestivum*) [103] and canola (*Brassica napus*) [106]. Motif 6 (CCGACGCG/CGCGTCGG) matches ABREs from maize [107], and rice [108]. Motif 7 (CCAACGCG/CGCGTTGG) matches an ABRE from barley [109] and rice [107]. Motifs 6, 8 (CACCGACC/GGTCGGTG) and 9 (TGGTCGGT/ACCGACCA) match low temperature response elements known as C-repeats (CRT, consensus sequence: RCCGAC), found in numerous species [110–112].

**Table 4:** Selected *de novo* motifs found in SK_n_ dehydrin promoters and their putative function identified through PLACE database.

	Match in PLACE^5^			
*De novo* motif	Sequence	Name	E-value^6^	Function
5. Seeder^1^ MCACGTGTC^**2**^ (120/120^3^, 9.2e-19^4^)	GGACACGTGGC	ABRETAEM	6.5e-13	ABRE found in wheat
	TGACACGTGGCA	HY5AT	2.4e-12	Bound by HY5, involved in light regulation of transcriptional activity
	TCCACGTGTC	SGBFGMGMAUX28	1.1e-13	Recognized by G-box binding factors in soybean. Found in auxin-responsive genes
	MCACGTGGC	GBOXLERBCS	8.1e-12	Sequence found in promoters of light-regulated genes
	CGCCACGTGTCC	ABREBNNAPA	2.4e-21	ABRE found in *Brassica napus*
6. MEME CCGACGCG (120/120, 1.2e-50)	CCCACGTGGC	ABREAZMRAB28	3.0e-05	ABRE, ABA and water-stress responses. Binding site of CBF2.
	GCCGCGTGGC	ABREMOTIFIIIOSRAB16B	4.0e-05	ABRE Motif III found in rice
	CCACGTGGCC	LTRECOREATCOR15	5.3e-06	LTRE
7. Seeder SCAACGCG (120/120, 2.0e-10)	TCCACGTCTC	ABRE3HVA1	2.3e-05	ABRE found in barley
	GCCGCGTGGC	ABREMOTIFIIIOSRAB16B	6.0e-05	ABRE Motif III found in rice
8. MEME CACCGACC (119/120, 3.8e-59)	RCCGAC	DRECRTCOREAT	1.9e-08	DRE/CRT found in genes expressed in response to cold and dehydration
	CCCACCTACC	ACIPVPAL2	3.8e-07	Required for vascular specific expression
	ACCGACA	LTREATLTI78	3.1e-07	LTRE
9. Seeder KKGTCGGY (120/120, 4.9e-07)	ACCGACA	LTREATLTI78	8.4e-07	LTRE
	RCCGAC	DRECRTCOREAT	2.4e-08	DRE/CRT found in genes expressed in response to cold and dehydration
10. MEME GTGGGVCC (61/120, 2.5e-22)	GGTCCCAT	GGTCCCATGMSAUR	8.3e-09	Auxin RE found in soybean
	CTCCCAC	BOXCPSAS1	3.9e-06	Involved in light-induced repression

^1^Number of the motif and the *de novo* discovery software that was used to locate that motif.

^2^Motif consensus sequence in IUPAC nucleotide code.

^3^Occurrence is the number of promoters containing a *de novo* motif out of the total number of promoters analyzed for a specific dehydrin class, presented in the parentheses.

^4^Siginificance of the motif, E-value calculated by MEME, Q-value calculated by Seeder, presented in the parentheses.

^5^PLACE matches were identified using STAMP, only significant matches with E-value < 0.05 are presented.

^6^E-value of the match with PLACE motif.

In addition, motif 5 matches an element from tomato and Arabidopsis light-regulated genes [113,114] and motif 10 (GTGGGACC) matches an element from pea involved in light-induced repression [115].

The presence of these significantly overrepresented motifs indicates that the SK_n_ dehydrins are regulated at the transcriptional level and their expression is modulated in response to cold and ABA. SK_n_ dehydrins should also be expressed in response to drought, since CRT, which is also called dehydration responsive element [116], is found in their promoters. The circadian clock controls cold induction of C-repeat binding factors (CBFs), which in turn bind CRT/DRE elements [117]. Phytochrome and cryptochrome genes are also regulated by a circadian clock in Arabidopsis [118]. COR27, a cold-induced gene, is regulated by circadian clock related evening elements (EE) [119]. In addition to EE, the COR27 promoter also contains multiple ABREs and G-boxes, to which motifs 5 and 6 also match. The core EE (AATATCT) [120] is found in 18 out of 73 SK_n_ gene promoters analyzed. Motifs involved in light-induced regulation of gene expression found in the promoters of SK_n_ genes could participate in modulation of these genes by the circadian clock. It has been shown previously, using bioinformatics methods, that the promoters of cold-regulated genes contain CRTs and ABREs [112,121] and our data also support those findings.

Motifs 5 and 10 match an auxin response element found in soybean GmAux28 [122] and SAUR15A promoter, respectively [123]. It has been shown previously that numerous genes related to auxin response in Arabidopsis are modulated in response to cold, such as auxin response factor ARF7 or the PINOID-binding protein 1 that is involved in hormone signaling and stress response [124].

### Y_n_SK_n_ dehydrins promoters contain multiple ABREs, light REs and a CRT

Y_n_SK_n_ dehydrins represent the largest subclass out of the five dehydrin classes analyzed. A total of 123 Y_n_SK_n_ gene promoters were analyzed (Table 1). The Y_n_SK_n_ dehydrins are expressed in response to ABA, dehydration and high salinity [3,5,100]. The two motifs presented in Table 5 and S2 Table (Motifs 11 and 12) match numerous elements in the PLACE database and both were discovered using a Seeder. Motif 11 (GACACGTGGC) is very similar to Motif 5 (GACACGTGT), found in the SK_n_ dehydrin promoters and they both match several of the same motifs in PLACE database, namely ABREs, G-box and light response elements. Motif 13 (CACCGAC) is almost identical to Motif 8 (CACCGACC) discovered in the SK_n_ dehydrin promoters, which matches CRT/DRE necessary for CBF mediated cold and dehydration response [116]. Overall, motifs found Y_n_SK_n_ dehydrin promoters are very similar to those found in SK_n_ dehydrin promoters indicating that they possibly have a similar function, and that these two classes may have diverged more recently than the other classes. While the function of the Y-segment in the gene products of Y_n_SK_n_ and Y_n_K_n_ dehydrins is not known, it shows similarity to the nucleotide binding domain of plant chaperones [125]. The gene products of the other dehydrin classes do not have any such domains. In addition, there are evolutive constraints on the Y-segment in a dehydrin from arctic *Oxytropis* species compared with temperate species [126], suggesting that the Y-segment might carry an important function that differentiates Y_n_SK_n_ from SK_n_ dehydrins. Some of the published data shows that Y_n_SK_n_ dehydrins are not expressed in response to cold [3,5], however there is evidence that after a period of acclimation they do accumulate in Red-Osier Dogwood (*Cornus sericea* L.) [127] and apple trees [128]. It is possible that cold-induced Y_n_SK_n_ dehydrin expression was not detected in some data sets due to a limited time of exposure to low temperature.

**Table 5:** Selected *de novo* motifs found in Y_n_SK_n_ dehydrin promoters and their putative function identified through PLACE database.

	Match in PLACE^5^			
*De novo* motif	Sequence	Name	E-value^6^	Function
11. Seeder^**1**^ SACACGTGG^**2**^ (123/123^3^, 4.8e-38^4^)	GGACACGTGGC	ABRETAEM	1.1e-16	ABRE found in wheat
	TCCACGTGTC	SGBFGMGMAUX28	2.5e-13	Recognized by G-box binding factors in soybean. Found in auxin-responsive genes
	CGCCACGTGTCC	ABREBNNAPA	6.7e-16	ABRE found in rapeseed
	TGACACGTGGCA	HY5AT	6.7e-16	Bound by HY5, involved in light regulation of transcriptional activity
	MCACGTGGC	GBOXLERBCS	7.3e-14	Sequence found in promoters of light-regulated genes
12. Seeder CRCCGAC (123/123, 3.1e-11)	RCCGAC	DRECRTCOREAT	2.3e-09	DRE/CRT found in gene expressed in response to cold and dehydration
	RYCGAC	CBFHV	1.4e-07	CRT found in barley (*Hordeum vulgare*)

^1^Number of the motif and the *de novo* discovery software that was used to locate that motif.

^2^Motif consensus sequence in IUPAC nucleotide code.

^3^Occurrence is the number of promoters containing a *de novo* motif out of the total number of promoters analyzed for a specific dehydrin class, presented in the parentheses.

^4^Siginificance of the motif, E-value calculated by MEME, Q-value calculated by Seeder, presented in the parentheses.

^5^PLACE matches were identified using STAMP, only significant matches with E-value < 0.05 are presented.

^6^E-value of the match with PLACE motif.

### Y_n_K_n_ dehydrins promoters contain ABREs and light REs

Y_n_K_n_ dehydrins represent the smallest subgroup, with only 21 members found in 51 plant genomes (Table 1). Y_n_K_n_ dehydrins are known to be expressed in response to cold [129,130], and two motifs were detected in their promoters (Table 6, S2 Table). One was identified using Seeder and the other using MEME. Both motifs match several ABREs and light REs in the PLACE database. Motif 13 (TAACACGTGTC/GACACGTGTTA) is similar to motif 11 (GACACGTGGC/GCCACGTGTC) identified in Y_n_SK_n_ dehydrins and it matches the some of the same motifs in PLACE. Motif 14 (ACGTGGCA/TGCCACGT) is similar to motif 11 (GACACGTGGC) found in Y_n_SK_n_ dehydrin. The lack of CRTs in the promoters of Y_n_K_n_ dehydrins suggests that they might be expressed in response to cold in ABA-dependent manner, not linked with the CBF transcription factors [18].

**Table 6:** Selected *de novo* motifs found in Y_n_K_n_ dehydrin promoters and their putative function identified through PLACE database.

	Match in PLACE^5^			
*De novo* motif	Sequence	Name	E-value^6^	Function
13. Seeder^1^ YRACACGTGTCC^2^ (21/21^3^, 1.5e-09^4^)	GGACACGTGGC	ABRETAEM	4.1-e11	ABRE found in wheat
	CGCCACGTGTCC	ABREBNNAPA	7.8e-11	ABRE found in rapeseed
	CGCACGTGTC	ABRE2HVA22	6.3e-09	ABRE2 found in barley HVA22 gene
	TCCACGTGTC	SGBFGMGMAUX28	6.3e-09	Recognized by G-box binding factors in soybean. Found in auxin-responsive genes
	AACGCGTGTC	CE3OSOSEM	1.1e-10	Coupling element 3 found in rice, required for ABA induced expression
	TGACACGTGGCA	HY5AT	7.5e-09	Bound by HY5, involved in light regulation of transcriptional activity
14. MEME ACGTGKCA (21/21, 8.3e-05)	ACGTGKC	ACGTABREMOTIFA2OSEM	1.3e-11	Core of ABRE in rice
	ACGTGGCA	LRENPCABE	5.7e-13	Positive light RE in tobacco
	YACGTGGC	ABREATCONSENSUS	7.4e-09	ABRE found in Arabidopsis
	RTACGTGGCR	ABADESI1	2.0e-11	ABRE and desiccation response in rice

^1^Number of the motif and the *de novo* discovery software that was used to locate that motif.

^2^Motif consensus sequence in IUPAC nucleotide code.

^3^Occurrence is the number of promoters containing a *de novo* motif out of the total number of promoters analyzed for a specific dehydrin class, presented in the parentheses.

^4^Siginificance of the motif, E-value calculated by MEME, Q-value calculated by Seeder, presented in the parentheses.

^5^PLACE matches were identified using STAMP, only significant matches with E-value < 0.05 are presented.

^6^E-value of the match with PLACE motif.

## Conclusions

Numerous dehydrins were identified in 51 plant genomes, many of which are not found in protein databases such as InterPro or PROSITE, or they are not annotated in Phytozome. The identified dehydrins were categorized into five subclasses based on the occurrence of conserved protein segments. Three *de novo* motif discovery software tools were used to find statistically significant overrepresented motifs in the promoters of each group of dehydrins. These motifs were matched to known *cis*-regulatory elements in the PLACE database to help explain the regulation of dehydrin expression in response to different environmental stimuli.

Dehydrins are expressed in response to multiple stress stimuli. Although there is overlap in expression triggers between dehydrin subclasses, there are differences in the pattern of expression. Some of the dehydrins are expressed constitutively in all tissues [3,5] and more specifically in seeds [131,132]. The presence of ABREs, CRTs and light REs in the promoters of Y_n_SK_n_ and SK_n_ dehydrins indicates that they could be expressed in response to dehydration and cold in both ABA-dependent and independent pathway and that this expression is modulated by light.

While Y_n_SK_n_ and SK_n_ dehydrin are found in most species, often in several copies, the other three subclasses are encountered less often. It is probable that they either have specialized functions or they are expressed together with Y_n_SK_n_ and SK_n_ dehydrins to increase the overall protective effect against dehydration. It is important to note that the number of discovered dehydrins is probably an underestimation due to incompleteness of genome assembly and errors inherent in sequencing.

Dehydrins play an important role in the survival of plants facing various stresses. Motifs matching *cis*-regulatory elements linked to both ABA-dependent and independent stress response pathways, as well as light response pathways were detected in dehydrins from many different plant families. The implication of this finding is that the regulation of dehydrins is conserved in the plant lineages included in this study and that stress-linked selection pressure preserved *cis*-regulatory elements in the promoters of dehydrins through stabilizing selection.

## Supporting Information

S1 Table Annotation and meta-data about the dehydrins included in the study. Each identified dehydrin was further analyzed by BLAST to find the closest match at NCBI GenBank non-reduntant database. The fields are 1. Species; 2. Gene; 3. Dehydrin subgroup; 4. BLAST top hit e-value; 5. BLAST top hit accession; 6. BLAST top hit description; 7. K-segment location; 8. Y-segment location; 9. S-segment location.
(CSV)

S2 Table Motif logos of motifs discovered in dehydrin promoters. Weblogos were made for each of the motifs identified by *de novo* motif discovery algorithms in five classes of dehydrin genes. The motif numbers correspond to the motif numbers in Tables 2–6.
(DOCX)
