# Supplementary material for: De Novo Regulatory Motif Discovery Identifies Significant Motifs in Promoters of Five Classes of Plant Dehydrin Genes
Source: PLoS One. 2015 Jun 26;10(6):e0129016. doi: 10.1371/journal.pone.0129016 (PMC4482647; doi:10.1371/journal.pone.0129016)
Supplement: S2 Table — Weblogos were made for each of the motifs identified by de novo motif discovery algorithms in five classes of dehydrin genes. The motif numbers correspond to the motif numbers in Tables 2–6. (DOCX) [file pone.0129016.s002.docx]

Supplemental Table 2. Motif logos of motifs discovered in dehydrin promoters

| **Motif number/**  **Software^1^** | **Dehydrin subclass^2^** | **Motif consensus^3^** | **Motif logo^4^** |
| --- | --- | --- | --- |
| 1. Seeder | KS | AWTCGGATAARR  YYTTATCCGAWT |    |
| 2. MEME | Kn | GGCMCCAC  GTGGKGCC |    |
| 3. MEME | Kn | AYGTCGGY  RCCGACRT |    |
| 4. Seeder | Kn | WNRCCGACAT  ATGTCGGYNW |    |
| 5. Seeder | SKn | MCACGTGTC  GACACGTGK |    |
| 6. MEME | SKn | CCGACGCG  CGCGTCGG |    |
| 7. Seeder | SKn | SCAACGCG  CGCGTTGS |    |
| 8. MEME | SKn | CACCGACC  GGTCGGTG |    |
| 9. Seeder | SKn | KKGTCGGY  RCCGACMM |    |
| 10. MEME | SKn | GTGGGVCC  GGNCCCAC |    |
| 11. Seeder | YnSKn | SACACGTGGC  GCCACGTGTS |    |
| 12. Seeder | YnSKn | CRCCGAC  GTCGGYG |    |
| 13 Seeder | YnKn | YRACACGTGTCC  GGACACGTGTYR |    |
| 14. MEME | YnKn | ACGTGKCA  TGMCACGT |    |

^1^Number of the motif and the *de novo* discovery software that was used to locate that motif

^2^One of 5 dehydrin subclasses in the promoters of which the listed motif was found

^3^Motif consensus sequence and its reverse complement (shown below) in IUPAC nucleotide code

^4^Motif logo representing the occurrence of a specific nucleotide at a respective position, top logo represents forward sequence and bottom logo represents its reverse complement. The x-axis represents the position of a nucleotide and the y-axis represent the amount of information in bits.
